# A functional promoter from the archaeon *Halobacterium salinarum* is also transcriptionally active in *E. coli*

**DOI:** 10.1186/s12866-022-02489-y

**Published:** 2022-03-24

**Authors:** Jinye Liang, Zhenghui Quan, Jianyu Zhu, Min Gan, Ping Shen

**Affiliations:** 1 Key laboratory of Biometallurgy, Ministry of Education, School of Minerals Processing and Bioengineering, Central South University, 410083 Changsha, China; 2 College of Life Sciences, Wuhan University, 430000 Wuhan, China

**Keywords:** Functional, Promoter, Archaea, -29 elements, *Escherichia coli*

## Abstract

**Background:**

Archaea form a third domain of life that is distinct from Bacteria and Eukarya. So far, many scholars have elucidated considerable details about the typical promoter architectures of the three domains of life. However, a functional promoter from the archaeon *Halobacterium salinarum* has never been studied in *Escherichia coli*.

**Results:**

This paper found that the promoter of *Halobacterium salinarum* showed a promoter function in *Escherichia coli*. This *Escherichia coli* promoter structure contains − 10 box, -10 box extension and − 29 elements, however, no -35 box. The − 29 element is exercised by the TATA box in archaea. And we isolated the RM10 fragment that possessed the fusion characteristics of bacteria and archaea, which was overlapped with functionality of TATA box and − 29 elements.

**Conclusions:**

The − 29 element reflects the evolutionary relationship between the archaeal promoter and the bacterial promoter. The result possibly indicated that there may be a certain internal connection between archaea and bacteria. We hypothesized that it provided a new viewpoint of the evolutionary relationship of archaea and other organisms.

**Supplementary Information:**

The online version contains supplementary material available at 10.1186/s12866-022-02489-y.

## Background

Archaea is a third domain of life different from bacteria and eukaryotes. Archaeal organisms have a unique position in the evolutionary tree of life, and share features in common with both bacteria and eukaryote. Therefore, comparative analyses of certain metabolic pathways (arginine metabolism and glycolysis [1]) or information transfer systems between archaea and the other two domains of life may provide useful clues for understanding the emergence and evolution of these pathways [2].

The core promoter architecture is such a example. Considerable details about the typical promoter architectures of the three domains of life (archaea, bacteria and eukarya) have been elucidated. It has now been confirmed that the core promoter architecture of archaea is closely similar to eukaryotic RNA polymerase II promoters, while sharing few similarities with the bacterial paradigm. Multiple functional and statistical analysis have identified three basal promoter elements common to the three archaea groups:an AT-rich TATA box centered around − 26/−27, which is the major basal promoter element, (2) an adjacent purine-rich region around − 33/−34, which is designated as transcription factor TFB, recognition element (BRE), and (3) a weakly conserved initiator element (INR) around the transcription start site. From the perspective of the sequence and spatial requirements, the archaeal promoter elements are homologous to those of eukarya (eukaryotic TATA box, BRE, and initiator elements). During the transcription initiation in archaea, the TATA box and BRE function as the binding sites for archaeal homologues of the eukaryotic TATA box binding protein (TBP) and TFIIB, the latter is called as TFB in archaea. The formed TBP-TFB-promoter ternary complex recruits the RNA polymerase to specifically initiate transcription in a eukaryotic-like manner [3, 4]. Notedly, the situation of bacteria is obviously different. The bacterial RNA polymerase holoenzyme is directly recruited to the promoter in a sequence specific manner. The DNA sequence elements responsible for this recognition have been intensively studied [5]. Four different sequence elements have been identified in σ70 (the predominant σ factor), which is dependent promoters in bacteria, including the − 10 box, the − 35 box, the extended − 10 element (a TG dinucleotide located immediately upstream of the − 10 box) and the UP element (a ~ 20 bp sequence located upstream of the − 35 box). In addition, bioinformatic analysis indicated that there are putative promoter elements with in − 29 ± 2 regions of a considerable portion of bacterial promoters [6, 7]. In bacteria, the combination of the above-mentioned promoter elements provides the basis for formation of multiple specific sequences for the initial binding of RNA polymerase, but the relative contribution of each element varies from promoter to promoter [8]. In general, any deficiency in one of these elements can be compensated by the other [9]. Therefore, according to the current knowledge, the two major types of promoter architectures of bacteria and archaea/eukaryotes seem to be very clear. They do not share a common ancestor or evolutionary connection.

However, our previous study [10] found such an archaeal promoter (*Haloarcula hispanica* amyH gene promoter), which is inconsistent with the established view.

The core promoter architecture of the halophilic archaeon *Haloarcula hispanica*’s amyH gene was revealed to possess a combination of the typical characteristics (both structural and functional) of archaeal and bacterial promoters. This suggested that the core promoters of some archaeal genes may share common features with their counterparts in bacteria [11].

Our occurrent study provides a new evidence for this hypothesis. In the present study, a promoter of the halophilic archaeon *Halobacterium salinarum* was shown to possess promoter activity in haloarchaea (archaea) as well as in *Escherichia coli* (bacteria). The core functional promoters responsible for the promoter activity in haloarchaea and *E. coli* overlap with each other, which may play a physiological function of binding to RNA polymerase. Thus, we speculated that some evolutionary relationships may exist between basal promoter structures of halophilic archaea and bacteria. A functional promoter from *Halobacterium salinarum* is transcriptionally active in *E. coli*, which may expand our understanding of the evolutionary history of transcription.

## Results

### A haloarchaea-origin promoter fragment exhibits promoter activity in both haloarchaea and *E. coli*

A plasmid genomic library of halophilic archaeon *Halobacterium salinarum* R1 was constructed by using the *E. coli* promoter probe vector pKK232-8 (see section Cloning of the RM10 promoter fragment from *Halobacterium salinarum*). The genomic DNA fragments of *H. salinarum* R1 were generated by SalI partial digestion and inserted upstream of the promoterless cat (chloramphenicol acetyltransferase) gene in pKK232-8. After transforming into *E. coli* DH5α, the resulting transformants were selected, and then their chloramphenicol resistance was determined on LB plates containing gradient chloramphenicol. Among these putative promoter clones, two clones showed chloramphenicol resistance above 100 µg/mL chloramphenicol, while *E. coli* DH5α containing pKK232-8 was merely sensitive to 5 µg/mL chloramphenicol. These results demonstrated that the corresponding haloarchaea–origin inserts (a 492-bp fragment was named as RM07 and a 1848-bp fragment was named as RM10) exhibited considerable promoter activity in *E. coli*, confirming the RM07 fragment and the RM10 fragment (GenBank accession no. AY640305) were worth for further investigation. The RM07 fragment has firstly been investigated [12] and revealed to possess a combination of the typical characteristics (both structural and functional) of archaeal and bacterial promoters. This research led to the hypothesis that the RM10 fragment is a similar type. This hypothesis was confirmed by promoter function analysis of haloarchaea promoter probe vector pSY2 and haloarchaea host strain *H. volcanii* WFD11. *H. volcanii* WFD11 possesses a genetic system very similar to other haloarchaea (including *H. salinarum*) and is widely used as the host organism in haloarchaea genetic analysis. Use a promoterless *bgaH* (haloarchaeal β-galactosidase) gene [13, 14] in pSY2 as the reporter gene for promoter activity assessment. The construct of pSY-P1848 has fused the upstream of RM10 of the promoterless bgaH gene in pSY2, and the plain vector pSY2 were transformed into *H. volcanii* WFD11, respectively, and then the obtained transformants were independently measured for intracellular β-galactosidase activity. The β-galactosidase activity in *H. volcanii* (pSY-P1848) was determined to 98.2 mU/mg of total cellular protein, while no β-galactosidase activity was detected in *H. volcanii* (pSY2), demonstrating that the haloarchaea-origin RM10 fragment possesses promoter activity in haloarchaea (Archaea) as well as in *E. coli* (Bacteria) as expected.

### Determination of the transcription start sites

**Fig. 1:**
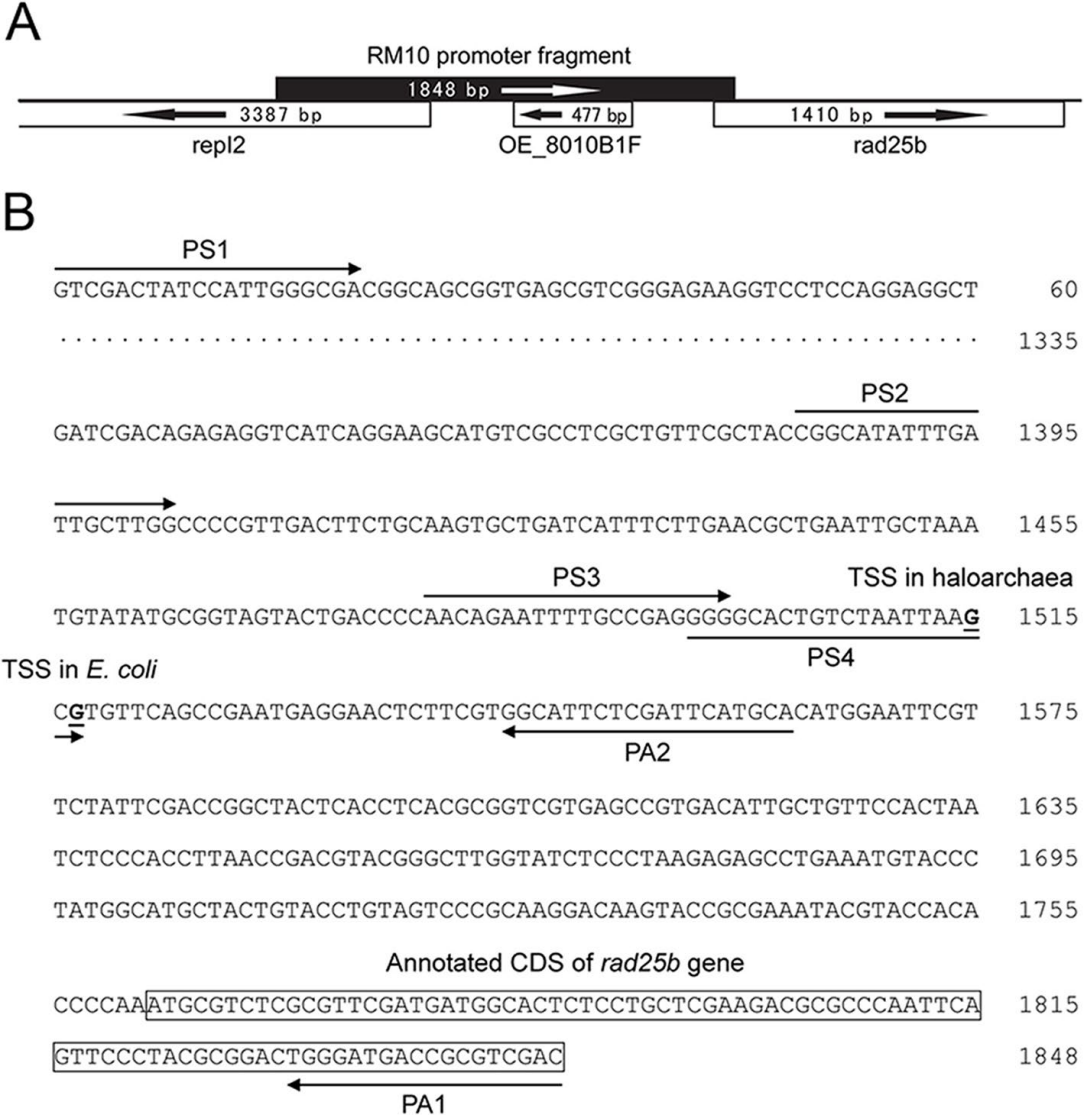
**A** the full-length (1848 bp) RM10 promoter fragment and **B** PS1, PS2, PS3, PS4, PA1, and PA2, with BamHI/HindIII sites incorporated at the 5’ ends of the forward/reverse primers in *E. coli*. (“PS1, PS2, PS3, PS4, PA1 and PA2” are the primers, “TSS in haloarchaea” means the transcription start sites of haloarchaea, “TSS in *E.coli”* indicates the transcription start sites of *E.coli*.)

**Figs. 2:**
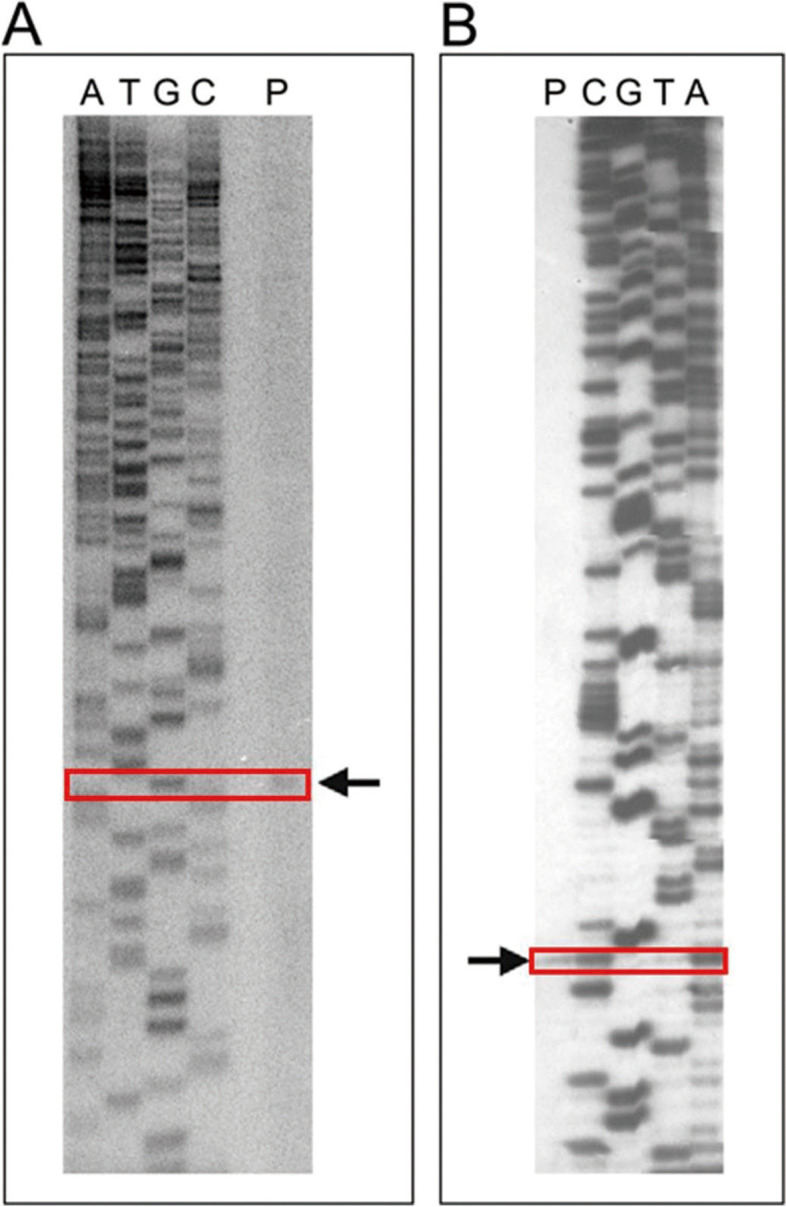
Primer extension analyses by RM10 fragment in **A** the haloarchaeal host strain (*H. volcanii WFD11*) and **B** *E.coli*. (“P” represents the Primer extension of promoter fragment.)

In order to locate the functional promoter in RM10 responsible for its promoter activity in haloarchaea, the full-length (1848 bp) RM10 fragment was sequenced (GenBank accession no. AY640305) and positioned in the genome of *H. salinarum* R1 (see Fig. 1(A). Primer extension analyses were performed to separately map the start sites of the transcriptions driven by RM10 fragment in the haloarchaea host strain (*H. volcanii* WFD11) and the *E.coli* (Fig. 2(A) and (B)). Total RNA of *H. volcanii* WFD11 containing plasmid pSY-P1848 was served as the templates. The RM10-driven transcripts in the haloarchaea organism was found to start from the guanine base (designated + 1(H)) 247 bp upstream of the ATG start codon of the rad25b gene as shown in Fig. 1(B). Further analysis of the nucleotide sequence upstream of this transcription start site (+ 1(H)), a typical TATA box (-30(H) TTTAAT − 25(H)) located 25 bp upstream of + 1(H), and a putative BRE (-35(H) AA -34(H)) upstream of the TATA box were identified. The above results demonstrate that the RM10 fragment contains one native promoter of *H. salinarum* R1 and this promoter is a gene of *H. salinarum* R1.

This speculation is quite conceivable, because an incomplete analysis of the *H. salinarum* R1’s transcriptome has been detected multiple new genes (including protein-coding genes and ncRNA-coding genes) that were previously missing from the original genome annotation of *H. salinarum* R1 [10].

Next, the start site of RM10 fragment in *E. coli* was determined by primer extension analysis (Fig. 2(B)), which was unequivocally located to a guanine (designated TSS in *E. coli*) 245 bp upstream of the rad25b start codon (just 2 bp downstream of the aforementioned TSS in haloarchaea) as shown in Fig. 1(B). The nucleotide sequence was further analyzed, the upstream of + 1(E) revealed a typical − 10 box (-11(E) TCTAAT − 6(E)) and a putative extended − 10 element (-13(E) TG -12(E)) located immediately upstream of the − 10 box, however, no sequence corresponding to the − 35 box was found.

Within the scope of RM10, the precise region of the functional *E. coli* promoter still needs to be further determined, but the 3’ boundary of the functional *E. coli* promoter was already set with the locating of + 1(E), which indicated that the core functional promoters responsible for the promoter activity in haloarchaea and *E. coli* overlap with each other.

### Identification of the sequences that control promoter activity in haloarchaea

**Fig. 3:**
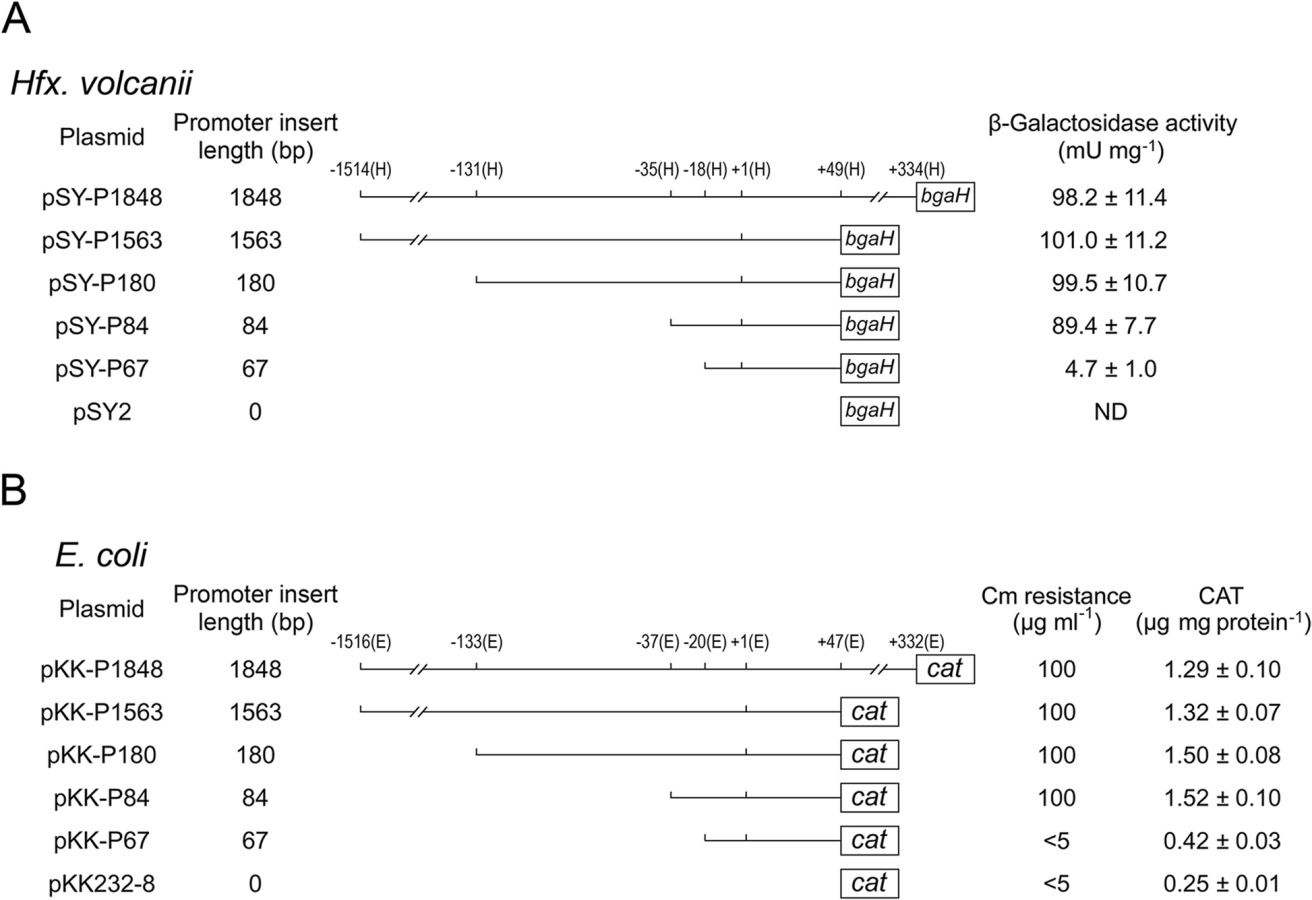
The RM10 fragment of the core structure of the inner haloarchaeal promoter from plasmid in *H. volcanii* (**A**) and *E. coli* (**B**). The reporter gene expression of each construct was determined as shown on the right. All results are shown as an average ± S.D. of three independent experiments. Cm, chloramphenicol. ND, no detectable β-galactosidase activity (< 0.5 mU/mg)

To thoroughly analyze the RM10 fragment and definitely locate the core structure of the inner haloarchaea promoter, a series of 5’ unidirectional deletion mutants were derived from pSY-P1848 (see section Cloning of the RM10 promoter fragment from *Halobacterium salinarum* and Fig. 3(A)) and measured for their abilities to drive the bgaH reporter gene in *H. volcanii*. The results show that the 5’-most region, -1514(H) to -36(H), and 3’-partial region, + 50(H) to + 334(H), can be removed without substantially altering promoter activity (Fig. 3(A)). In contrast, further removal of the sequence from − 35(H) to -19(H) will lead to a drastic abolishment of promoter function (Fig. 3(A)), suggesting that the core structure of the inner haloarchaea promoter is located downstream of position − 35(H).

**Fig. 4:**
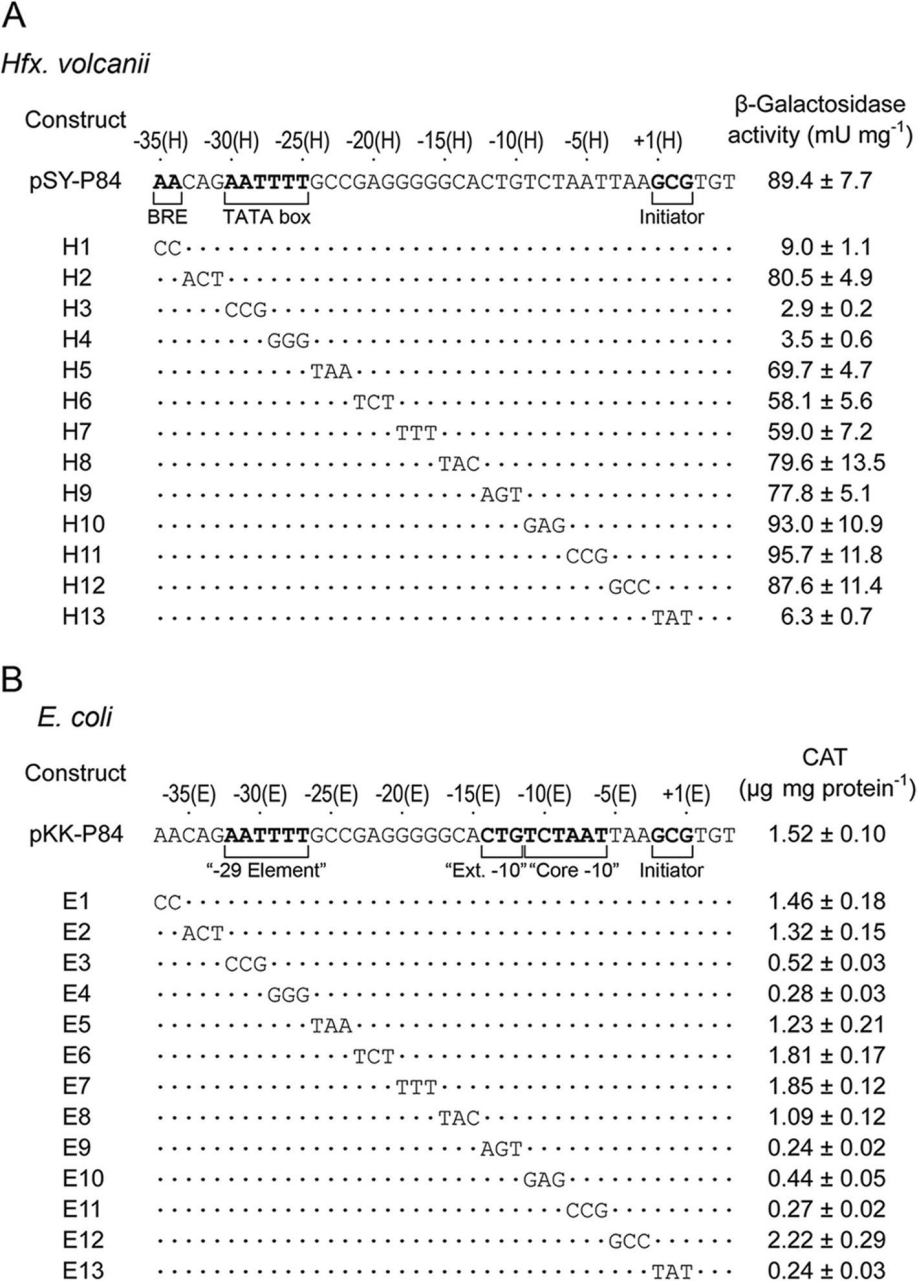
The essential sequence elements of the inner haloarchaea promoter of the RM10 fragment between − 35 and + 3 were performed in in *H. volcanii* (**A**) and *E. coli* (**B**). The β-galactosidase and CAT activity of construct were determined and was shown on the right of the corresponding template sequence. All results are shown as an average ± S.D. of three independent experiments

The essential sequence elements of the inner haloarchaea promoter were more accurately defined, a 3 bp scanning mutagenesis spanning a 38 bp region of the RM10 fragment between − 35(H) and + 3(H) was performed, using the wild-type promoter fragment − 35(H) to + 49(H) as a template (Fig. 4(A)). All the base substitutions were transversions rather than transitions. The various mutant templates were generated and analyzed for promoter activity as mentioned above (Fig. 4(A)). Four mutants (H1, H3, H4 and H13) showed drastically reduce promoter activities compared with the wild-type promoter template, identifying three discrete regions essential for promoter function: -35(H) to -34(H), -30(H) to -25(H), and + 1(H) to + 3(H) (Fig. 4(A), in bold), which is corresponding to the BRE, TATA box and initiator elements, respectively. All these results demonstrate that the presumed haloarchaea promoter structure (the putative BRE, TATA box and initiator elements) is indeed the core structure of the haloarchaea promoter encompassed in RM10.

### Identification of the sequences that control promoter activity in *E. coli*

**Fig. 5:**
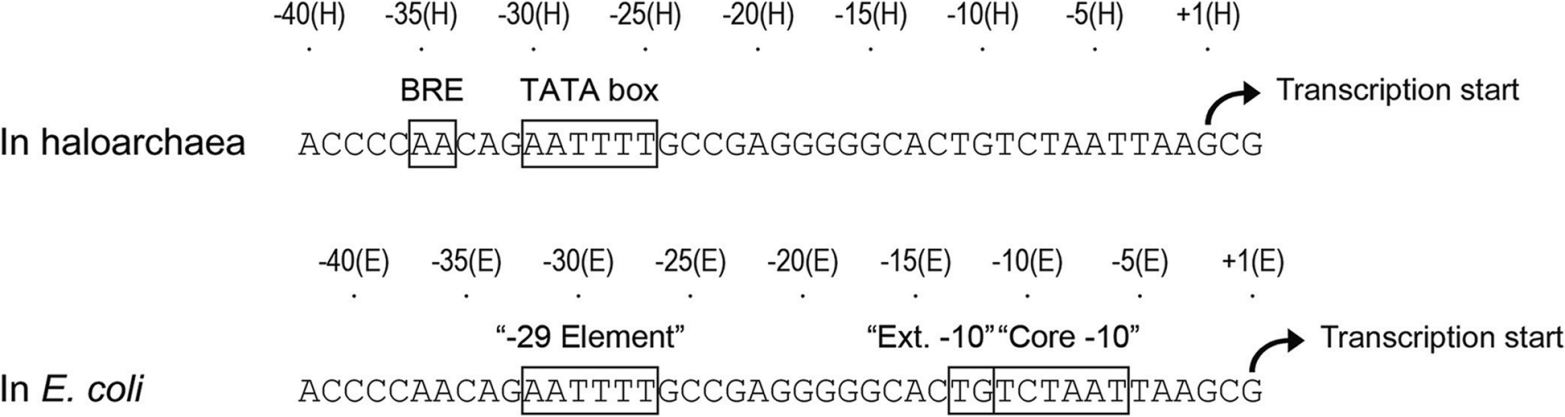
The sequence − 35(E) to -25(E) of *E. coli* is named the “-29 element” and functional elements were shown in haloarchaea and *E. coli*, respectively

In order to identify the sequences required for promoter activity in *E. coli*, the RM10 fragment was subjected to sequential mutation analysis again by using the *E. coli* promoter probe vector pKK232-8. As a first step, a series of sequential 5’ deletion was introduced into the RM10 fragment (see section Construction of deletion mutants for reporter gene analyses in *H. volcanii* and *E*. *coli* and Fig. 3(B)). The generated deletion mutants were tested for their abilities to drive the CAT gene expression in *E. coli.* The results show that the region between − 37(E) and + 47(E) is the major control responsible for the promoter function of RM10 in *E. coli* (Fig. 3(B)). Subsequently, a 3 bp scanning mutagenesis spanning a 38 bp region of the RM10 fragment between − 37(E) and + 1(E) was performed, using the wild-type promoter fragment − 37(E) to + 47(E) as templates (Fig. 4(B)). The various mutant templates were generated and assayed for promoter activities as mentioned above (Fig. 4(B)). Six mutants (E3, E4, E9, E10, E11 and E13) showed severely decline promoter activities compared with the wild-type promoter template, identifying three discrete regions essential for promoter function: -32(E) to -27(E), -14(E) to -6(E), and − 2(E) to + 1(E) (Fig. 4(B), in bold). The region − 14(E) to -6(E) encompasses the conserved − 10 box and extended − 10 element defined by location and sequence (Fig. 4(B)). The region from − 2(E) to + 1(E) corresponds to the initiator element. The functional region − 32(E) to -27(E) is an interesting discovery, since no bacterial promoter has been reported previously to contain functional element around this position. However, as described in the introduction section, two previous bioinformatic studies [7, 15] have indicated the presence of a putative promoter element within − 29 ± 2 regions of quite a portion of bacterial promoters. Thus, the sequence − 32(E) to -27(E) is designated as the “-29 element” (Fig. 5). In addition, it is also fascinating that this hexamer shares exactly the same six nucleotides with the haloarchaea TATA box defined above.

## Discussion

The promoter function between halophilic archaea and *E. coli* depended on a common sequence element. This discovery is similar to results of our previous studies, but it is different from the traditional understanding of the basic bacterial promoter structure. This archaeal promoter functional analysis revealed that archaeal promoter existed a typical structure of an archaea promoter, while it had an atypical structure of an *E. coli* promoter. Promoter fragment with *E. coli* structure confers the function of this promoter in *E. coli*. But it also had an atypical that this *E. coli* promoter structure consists of -10 box, -10 box extension and − 29 elements. Regrettably, no -35 box was found in this promoter. In fact, lots of *E. coli* σ70 promoters have been found to lack a -35 box, meanwhile, there is a -10 box extension [16]. The − 29 element is an interesting discovery, because there are currently no experiments to confirm the presence of functional elements around − 29 region (Fig. 5). Yet, two bioinformatics papers [6, 7] in 1997 and 1999 had predicted the presence of a conserved functional − 29 element in a portion of the *E. coli* promoters. The *E. coli* promoter containing the − 29 element is generally deficient in -35 box. In archaea, the physiological function of binding RNA polymerase is performed by the TATA box. Simultaneously, it is noted that the TATA box and − 29 elements in RM10 fragment overlaps with each other. Therefore, we speculate that this sequence element may reflect the evolutionary relationship of the archaeal promoter and the bacterial promoter. It is even said that this sequence element may exist in the basic transcriptional signal of the common ancestor, before the differentiation of bacteria and archaea/eukaryotes.

In conclusion, we separated the RM10 fragment that is very particular. The RM10 possessed promoter activity in haloarchaea (Archaea) as well as in *E. coli* (Bacteria), which was similar to the promoter of *H. hispanica* amyH. The promoter structure of RM10 fragment existed the fusion characteristics of bacteria and archaea [17]. All these results suggested that there may be some evolutionary correlation between the basic transcriptional signals of bacteria and archaea [18, 19]. In recent years, it has been found that RNA polymerases of three domains of life share similarities in structure and function [20]. Besides, almost all eukaryotic transcription currently relies on basic transcription factors. Therefore, we inferred that archaea and prokaryotes may existed a certain internal connection.

## Conclusions

In this research, We deduced that a Haloarchaea promoter (RM10 fragment) was shown promoter function in *E. coli*. Meanwhile, the promoter structure consisted of -10 boxes, -10 box extensions and − 29 elements, without − 35 boxes. The − 29 element overlapped with the TATA box required for the function of archaea and also performed the physiological function of binding with RNA polymerase. We hypothesized that it provided a new viewpoint of the evolutionary relationship of archaea and other organisms. A functional promoter from *Halobacterium salinarum*(archaea) was transcriptionally active in *E. coli*(bacteria), indicating that more investigations about archaea promoter activity are required in the future.

## Materials and methods

### Strains and culture conditions

*Halobacterium salinarum* R1 was grown in modified growth medium (25% MGM) [21]. *Haloferax volcanii* strain WFD11 was grown in modified growth medium (18% MGM, including NaCl 144 g, MgSO_4_·7H_2_O 21 g,·MgCl_2_·6H_2_O 18 g, KCl 4.2 g, CaCl_2_ 0.5 g, tryptone 5 g, yeast extract 1 g, 1 M Tris-HCl(pH = 7.2) 50 mL and H_2_O 1000 mL) [21], The strains were cultured on a shaker at 37 °C for 1 ~ 2 days. The medium was supplemented with 0.3 µg/mL novobiocin to select transformants. *Escherichia coli* strains DH5α and *Escherichia coli* strains JM110 were cultured in Luria-Bertani (LB) medium supplemented with antibiotics when necessary [22].

### Cloning of the RM10 promoter fragment from *Halobacterium salinarum*

The genomic DNA of *Halobacterium salinarum* R1 was isolated from [23] and partially digested by SalI. The obtained fragments were connected to the SalI site of the *E. coli* promoter probe vector pKK232-8 [24] upstream of a promoterless cat (chloramphenicol acetyltransferase) gene. The ligation products were transformed into *E. coli* DH5α, and 17 colonies were obtained on selective plates containing 20 µg/mL chloramphenicol. Then the chloramphenicol resistance of all transformants was determined independently by the plate streak method. Two of these clones were resistant to chloramphenicol above 100 µg/mL chloramphenicol, and the corresponding inserted fragments (named as RM07 and RM10) were characterized in our previous study [10] and present study, respectively.

### RNA isolations and primer extension analyses

Primer extension analyses were performed to separately map the start sites of the transcriptions, which were derived by RM10 fragment in the two host organisms (*H. volcanii* WFD11 and *E. coli* DH5α). According to the method described in [25], total RNA were isolated from *H. salinarum* and *H. volcanii* containing plasmid pSY-P1848 (see section Construction of deletion mutants for reporter gene analyses in *H. volcanii*, E. *coli* and Supplementary Information). Total RNA was also isolated from *E. coli* containing plasmid pKK-P1848(see section Construction of deletion mutants for reporter gene analyses in *H. volcanii*,*E. coli* and Supplementary Information ) by using the hot phenol method [26]. As described previously in paper [26], in each primer extension analysis, the total RNA sample was subjected to reverse transcription, using a 5’-32P-labeled antisense primer specific to the 3’ end sequence of the RM10 fragment. The sequences of the primers used are: HSRT, 5’-GGTCGAATAGAACGAACGAATTCCA-3’ (for the RNA sample from *H. salinarum*); HVRT, 5’-AGTAGCCGGTCGAATAGAA-3’ (for the RNA sample from *H. volcanii* containing plasmid pSY-P1848); ERT, 5’-CGAGAATGCCACGAAGAG-3’ (for the RNA sample from *E. coli* containing plasmid pKK-P1848). The primer extension products were analyzed on 8% polyacrylamide/urea sequencing gels. The transcription start sites were identified by the sequencing ladders directly parallel to the run-off reverse transcripts.

### Construction of deletion mutants for reporter gene analyses in *H. volcanii* and *E. coli*

To facilitate promoter analysis in haloarchaea, the *E. coli-H. volcanii* shuttle vector pSY2 was constructed based on pSY1 [17]. Use the BglII and NdeI restriction sites in pSY2 to introduce different promoter fragments before the promoterless bgaH (haloarchaeal β-galactosidase) reporter gene. The 1848 bp (full-length) RM10 fragment was used as a template to generate a series of deletion derivatives by PCR. The primers (PS1, PS2, PS3, PS4, PA1, and PA2, with BglII/NdeI sites incorporated at the 5’ ends of the forward/reverse primers respectively) are indicated in Fig. 1(B). The 1848, 1563, 180, 84 and 67 bp putative promoter fragments were amplified and cloned into the BglII/NdeI sites of pSY2, in order to generate constructs of pSY-P1848, pSY-P1563, pSY-P180, pSY-P84, and pSY-P67, respectively. All these reporter plasmids have been sequenced and verified, independently transformed into *H. volcanii* WFD11 through *E. coli* JM110 (dam-) as described previously [27].

The RM10 fragment was also subjected to deletion analysis in *E. coli* by using the *E. coli* promoter probe vector pKK232-8. The 1848 bp (full-length) RM10 fragment was again used as a template to generate a series of deletion derivatives by PCR. The primers (PS1, PS2, PS3, PS4, PA1, and PA2, with BamHI/HindIII sites incorporated at the 5’ ends of the forward/reverse primers respectively) are indicated in Fig. 1(B). The 1848, 1563, 180, 84, and 67 bp putative promoter fragments were amplified and cloned into the BamHI/HindIII sites of pKK232-8 upstream of the promoterless cat gene, generating constructs of pKK-P1848, pKK-P1563, pKK-P180, pKK-P84, and pKK-P67, respectively. All these reporter plasmids have been sequenced and verified, and independently transformed into *E. coli* DH5α for promoter activity assay.

### Construction of scanning mutants for reporter gene analyses in *H. volcanii* and *E. coli*.

A series of scanning mutations spanned a 38 bp region of the RM10 fragment from − 35 to + 3, which were relative to the experimentally determined transcription start site in haloarchaea (designated + 1(H) as described in section 4), were created to use the pSY-P84 construct as a template. The various 84 bp promoter fragments containing the desired mutations (with BglII/NdeI sites incorporated at the 5’/3’ ends) were commercially synthesized by AuGCT DNA-SYN Biotechnology Co., Ltd. (Beijing, China), and they were used to substitute the 84 bp wild-type promoter insert in construct pSY-P84, generating various mutant constructs as shown in Fig. 4(A). All constructed structures have been sequenced and verified, and independently transformed into *H. volcanii* WFD11 as described in section (Construction of deletion mutants for reporter gene analyses in *H. volcanii* and *E. coli)*.

A series of scanning mutations spanned a 38 bp region of the RM10 fragment from − 37 to + 1, which were relative to the experimentally determined transcription start site in *E. coli* (designated + 1(E) as described in section 4), were created to use the pKK-P84 construct as a template. The various 84 bp promoter fragments containing the desired mutations (with BamHI/HindIII sites incorporated at the 5’/3’ ends) were commercially synthesized by Invitrogen (Shanghai, China) and they were used to substitute the 84 bp wild-type promoter insert in construct pKK-P84, generating various mutant constructs as shown in Fig. 4(B). All constructed structures have been sequenced and verified, and independently transformed into *E. coli* DH5α for promoter activity assay.

### β-Galactosidase assays

β-Galactosidase activity was measured in cell lysates of relevant *H. volcanii* transformants by the ONPG assay as described by [10]. A certain amount of cell lysate (ultrasonic breaking) was added to the freshly prepared Assay buffer, after mixed thoroughly, and then ONPG was added to the reaction and the reactive temperature at 30 °C. The blank control was the Assay buffer without cell lysate. One unit of β-galactosidase activity (U) is defined as the amount of enzyme required to catalyze the hydrolysis of 1 µmol/min ONPG. The molecular absorbance coefficient of ONPG was measured at 405 nm. Each β-galactosidase activity value was normalized with the protein concentration of the corresponding cell lysate as measured by the Bradford method(0.1 mL sample and 5 mL dye solution were mix well, and then solutions were stranded at room temperature for 2 min, and absorbance was measured at 595 nm). All results are shown as an average ± S.D. of three independent experiments.

### CAT assays

In order to determine the expression of CAT, cultures of relevant *E. coli* transformants were grown in LB medium with an OD600 nm of 0.3 ~ 0.4. Samples were collected, after pelleting, the cells were resuspended in 50 mM Tris-HCl (pH = 7.5) and lysed by sonication. The cell extracts were centrifuged to remove intact cells and debris, and then the supernatants were used for CAT and total protein determinations. The CAT levels in cell extracts were determined by using the CAT ELISA kit (Roche) according to the manufacturer’s instructions. The total protein concentrations were determined by the Bradford method with BSA as a standard [28]. CAT concentrations were calculated as microgram per milligram of total protein. All results are shown as an average ± S.D. of three independent experiments.

## Supplementary Information

**Additional file 1.**

## Data Availability

The datasets generated and/or analyzed during the current study are not publicly available. If this paper are published, the data will be used. The datasets generated and/or analyzed during the current study are available in the NCBI website, which is in GenBank accession no. AY640305. Persistent web link or accession number to datasets as follows: https://www.ncbi.nlm.nih.gov/nuccore/AY640305.1/.
