# Supplementary material for: A functional promoter from the archaeon Halobacterium salinarum is also transcriptionally active in E. coli
Source: BMC Microbiol. 2022 Mar 24;22:79. doi: 10.1186/s12866-022-02489-y (PMC8943987; doi:10.1186/s12866-022-02489-y)

Supplementary Information

A functional promoter from the archaeon *Halobacterium salinarum* is also transcriptionally active in *E. coli*

Jinye Liang[1]† Zhenghui Quan[2]†, Jianyu Zhu[1,2*], Min Gan[1*], Ping Shen[2*]

1 Key laboratory of Biometallurgy, Ministry of Education, School of Minerals Processing and Bioengineering, Central South University, 410083, Changsha, China

2 College of Life Sciences Wuhan University, 430000, Wuhan, China

†Jinye Liang and Zhenghui Quan have contributed equally to this work.

***Correspondence:**

Corresponding Author: Jianyu Zhu*, zhujy@csu.edu.cn，Min Gan*, ganmin0803@sina.com and Ping Shen*, pingshen@whu.edu.cn.

Use the promoter reporter probe plasmid pKK232-8 to construct the recombinant plasmid containing the fragment RM10 and transform into *E. coli* DH5α to extract total RNA.

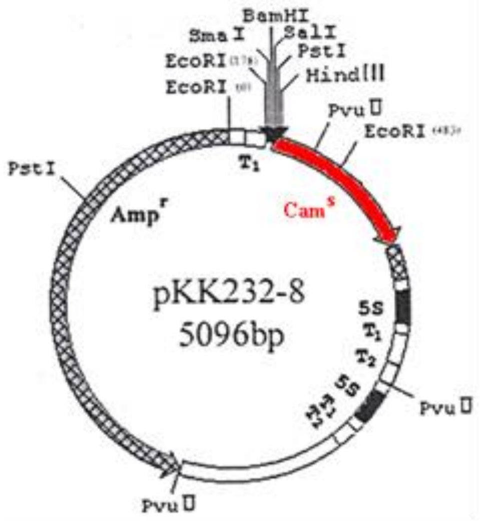

Figure S1-1 The map of the promoter probe plasmid pKK232-8, Amp^r represents ammonia resistance; Cam^r stands for chloramphenicol resistance.

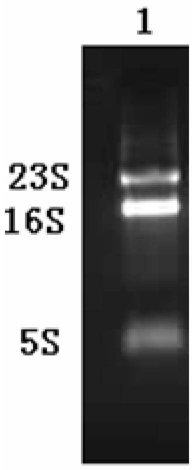

Figure S1-2 The 2% agarose gel electrophoresis diagram of total RNA of *E. coli* DH5α containing RM10 fragment in mid-log phase. Lane 1 is the total RNA extracted.

Two pairs of primers (Qrtf:5'-tcarcgctctggagtgaatacc-3' and Qrtr: 5'-ccgcttattatcacttattcagg-3') and (Qrtf:5'-tcarcgctctggagtgaatacc-3' and pCATR2: 5'-tgaaactcacccagggattg-3') were design for analysis to for analysis to detect the quality and purity of RNA and confirm the presence of the reporter gene cat mRNA in the extracted total RNA. Specific 422bp and 138bp RT-PCR product bands of predetermined size were detected in the total RNA, however, no specific PCR product

bands appeared in the negative control.

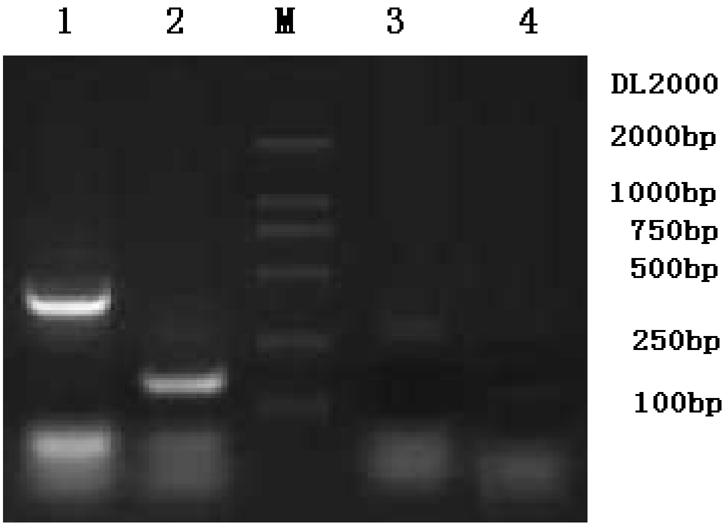

Figure S1-3 Agarose gel electrophoresis of RT-PCR products of the total RNA of E. coli DH5α containing RM10 fragment. Lane 1: RT-PCR product was used the total RNA as a template, Qrtf and Qrtr as primers. Lane 2: RT-PCR product was used total RNA as template, Qrtf and pCATR2 as primers. Lane 3: A direct PCR product was total RNA as a template, Qrtf and Qrtr as primers. Lane 4: The PCR product was used total RNA as template, Qrtf and pCATR2 as primers.

The *H. volcanii* WFD11 / *E. coli* DH5α shuttle plasmid pSY1 was used to construct a recombinant plasmid containing the RM10 fragment, and transformed the *H. volcanii* WFD11 and extract the total RNA.

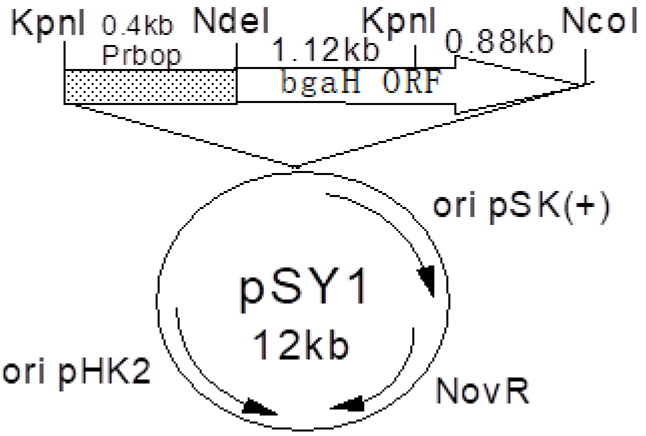

Figure S2-1 Structure of *H. volcanii* WFD11 / *E. coli* DH5α shuttle plasmid pSY1. *bgaH*: gene encoding extreme halophilic bacteria β-galactosidase; *Pr* bop: bacterial rhodopsin gene promoter; *ori* pHK2: *H. volcanii* WFD11 plasmid pHK2 replication region; *ori* pSK(+): *E. coli* DH5α plasmid pSK(+) replication region; NovR: novobiocin resistance gene.

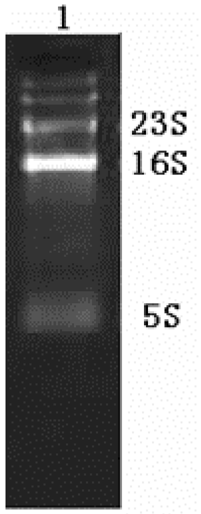

Figure S2-3 The 2% agarose gel electrophoresis diagram of the total RNA of *H. volcanii* WFD11 containing the RM10 fragment in the mid-log phase. Lane 1 represents the total RNA extracted.

A pair of primers (PSF5: 5'-tgcggtaccaccttaaccgacgtacgg-3'; haloRT: 5'-taaaccgttcgtaggc-3') were used for RT-PCR analysis to determine the presence of the reporter gene bgaH mRNA in the extracted total RNA and detect the quality of the RNA and purity.

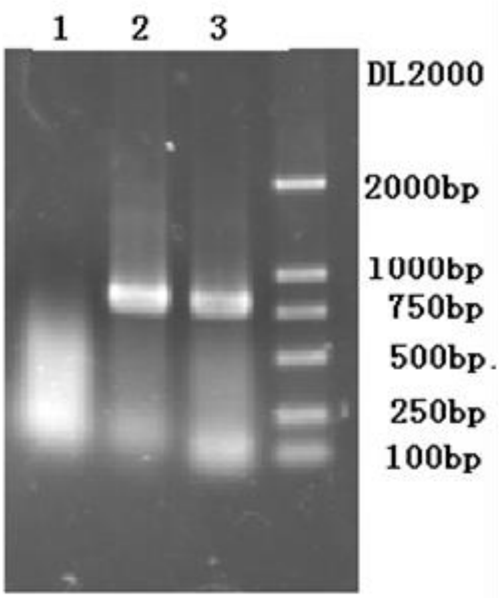

Figure S2-4 Agarose gel electrophoresis of RT-PCR products. Lane 1: the PCR product of PSF5 and haloRT with total RNA as template is used as a negative control. Lane 2: RT-PCR products of PSF5 and haloRT using total RNA as template. Lane 3: Directly use pSY1-10-F2 plasmid as a template for the PCR product of the positive control.

Halobacterium salinarum strain R1 clone 10 genomic sequence

GenBank: AY640305.1

LOCUS AY640305 1848 bp DNA linear
BCT 30-NOV-2004
DEFINITION *Halobacterium salinarum* strain R1 clone 10 genomic sequence.
ACCESSION AY640305
VERSION AY640305.1
KEYWORDS .
SOURCE *Halobacterium salinarum*
ORGANISM *Halobacterium salinarum*
Archaea; Euryarchaeota; Stenosarchaea group; Halobacteria;
Halobacteriales; Halobacteriaceae; Halobacterium.
REFERENCE 1 (bases 1 to 1848)
AUTHORS Zhu,J., Huang,Y. and Shen,P.
TITLE Study on Sequence Characteristic and Function of Promoter Activity
DNA fragment from *Halobacterium halobium* R1
JOURNAL Unpublished
REFERENCE 2 (bases 1 to 1848)
AUTHORS Zhu,J., Huang,Y. and Shen,P.
TITLE Direct Submission
JOURNAL Submitted (28-MAY-2004) College of Life Sciences, Wuhan
University,
Wuchang Luojiashan, Wuhan, Hubei 430072, P.R.China
FEATURES Location/Qualifiers
source 1..1848
/organism="Halobacterium salinarum"
/mol_type="genomic DNA"
/strain="R1"
/db_xref="taxon:2242"
/clone="10"
ORIGIN
1 gtcgactatc cattgggcga cggcagcggt gagcgtcggg agaaggtcct ccaggaggct
61 gggaatccag cgccgctact ggggttggag cccacgatag acgaccgtcg tcgagagaat
121 ccgtctggcc gtcctgctct ggcggaatac gcgaaattcc agaatcggcg gccacaggaa
181 gcgggattcc acgaaattcg aaggagttcg tgtgctcgga ttccagttcc tccatccccg
241 aggaagatga ggaggacgtc gtcgattcat cgttggcgga gaggagatcg gcggcgtcgt
301 cagcgccgat gtggcggagg actgccgcct cgtcgatgag cgacgactgc gcggcgtcct
361 cgagagagtc gtagtcggca agagcgtcgt caacggtggc gcggtactgg tcgcgagcac
421 gtgggtcgct ccggctggcg aggatctggg tgcgcttctc gacgaacgcg tcgcggtccg
481 gggcctcggc gaacttgcgg agtgtgcgag tcctgtaggt agtccgacag cgagggcagt
541 ctgtggtctt gcggccggcg gcggtgaacc agagctcgct cagtgcgagc aaccgacgag
601 ccagatggtg tggtgtccgg acataggcgt ctgtccggga agttgcgtgc tgacgactcc

661 cgagttagtc gtcgtacccg agaccgttcg cgttcgagga cagccgctgg tgactctcgc
721 caagagagcc acggagccac ggctaagggc gaaagctatt atgtcatggg cccgcgagaa
781 ccagatggtt gatcgggttc tcttgccgtc gggactagcc atcctcggcg gtgaaaccaa
841 ttatttcacc gtccagaggc atcccggacg attaggcacg tcgtgcggtt agcctacctt
901 ggttgcgtag cctttgccac cccgggcata aaaagttgtg tcggacatct ctcggtcact
961 ttttgcgtga atcgccgaca ggcacgtcgc catactccgg acaggagacc cagccttggc
1021 caaaagcagc gccaaatccc tggtctggcc gccactcatg ttcctcgcca caattcgggc
1081 actccacgaa cggcgggtca atcgcaaccg cctcttgttc catgtagtgg tttccttggt
1141 atgcctcgca gtgctggcag acgtttcccc acacctcttt ttcaagcgtg ttgctgtaga
1201 cacgttcgac aggagtatcg tactttgcaa gcacgccgcc aataggggtg ttgagatggt
1261 cgtcgcgagg ccagactatt ggcgtttgct caccgcactg ccagcactcg gtctgccacg
1321 tcagtaactt gtcttgatcg acagagaggt catcaggaag catgtcgcct cgctgttcgc
1381 taccggcata tttgattgct tggccccgtt gacttctgca agtgctgatc atttcttgaa
1441 cgctgaattg ctaaatgtat atgcggtagt actgacccca acagaatttt gccgaggggg
1501 cactgtctaa ttaagcgtgt tcagccgaat gaggaactct tcgtggcatt ctcgattcat
1561 gcacatggaa ttcgttctat tcgaccggct actcacctca cgcggtcgtg agccgtgaca
1621 ttgctgttcc actaatctcc caccttaacc gacgtacggg cttggtatct ccctaagaga
1681 gcctgaaatg taccctatgg catgctactg tacctgtagt cccgcaagga caagtaccgc
1741 gaaatacgta ccacacccca aatgcgtctc gcgttcgatg atggcactct cctgctcgaa
1801 gacgcgccca attcagttcc ctacgcggac tgggatgacc gcgtcgac

>AY640305.1 *Halobacterium salinarum* strain R1 clone 10 genomic sequence
GTCGACTATCCATTGGGCGACGGCAGCGGTGAGCGTCGGGAGAAGGTCCT
CCAGGAGGCTGGGAATCCAG
CGCCGCTACTGGGGTTGGAGCCCACGATAGACGACCGTCGTCGAGAGAAT
CCGTCTGGCCGTCCTGCTCT
GGCGGAATACGCGAAATTCCAGAATCGGCGGCCACAGGAAGCGGGATTCC
ACGAAATTCGAAGGAGTTCG
TGTGCTCGGATTCCAGTTCCTCCATCCCCGAGGAAGATGAGGAGGACGTCG
TCGATTCATCGTTGGCGGA
GAGGAGATCGGCGGCGTCGTCAGCGCCGATGTGGCGGAGGACTGCCGCCT
CGTCGATGAGCGACGACTGC
GCGGCGTCCTCGAGAGAGTCGTAGTCGGCAAGAGCGTCGTCAACGGTGGC
GCGGTACTGGTCGCGAGCAC
GTGGGTCGCTCCGGCTGGCGAGGATCTGGGTGCGCTTCTCGACGAACGCG
TCGCGGTCCGGGGCCTCGGC
GAACTTGCGGAGTGTGCGAGTCCTGTAGGTAGTCCGACAGCGAGGGCAGT
CTGTGGTCTTGCGGCCGGCG
GCGGTGAACCAGAGCTCGCTCAGTGCGAGCAACCGACGAGCCAGATGGTG
TGGTGTCCGGACATAGGCGT
CTGTCCGGGAAGTTGCGTGCTGACGACTCCCGAGTTAGTCGTCGTACCCGA
GACCGTTCGCGTTCGAGGA
CAGCCGCTGGTGACTCTCGCCAAGAGAGCCACGGAGCCACGGCTAAGGGC

GAAAGCTATTATGTCATGGG
CCCGCGAGAACCAGATGGTTGATCGGGTTCTCTTGCCGTCGGGACTAGCCA
TCCTCGGCGGTGAAACCAA
TTATTTCACCGTCCAGAGGCATCCCGGACGATTAGGCACGTCGTGCGGTTA
GCCTACCTTGGTTGCGTAG
CCTTTGCCACCCCGGGCATAAAAAGTTGTGTCGGACATCTCTCGGTCACTT
TTTGCGTGAATCGCCGACA
GGCACGTCGCCATACTCCGGACAGGAGACCCAGCCTTGGCCAAAAGCAGC
GCCAAATCCCTGGTCTGGCC
GCCACTCATGTTCCTCGCCACAATTCGGGCACTCCACGAACGGCGGGTCAA
TCGCAACCGCCTCTTGTTC
CATGTAGTGGTTTCCTTGGTATGCCTCGCAGTGCTGGCAGACGTTTCCCCAC
ACCTCTTTTTCAAGCGTG
TTGCTGTAGACACGTTCGACAGGAGTATCGTACTTTGCAAGCACGCCGCCA
ATAGGGGTGTTGAGATGGT
CGTCGCGAGGCCAGACTATTGGCGTTTGCTCACCGCACTGCCAGCACTCGG
TCTGCCACGTCAGTAACTT
GTCTTGATCGACAGAGAGGTCATCAGGAAGCATGTCGCCTCGCTGTTCGCT
ACCGGCATATTTGATTGCT
TGGCCCCGTTGACTTCTGCAAGTGCTGATCATTTCTTGAACGCTGAATTGCT
AAATGTATATGCGGTAGT
ACTGACCCCAACAGAATTTTGCCGAGGGGGCACTGTCTAATTAAGCGTGTT
CAGCCGAATGAGGAACTCT
TCGTGGCATTCTCGATTCATGCACATGGAATTCGTTCTATTCGACCGGCTAC
TCACCTCACGCGGTCGTG
AGCCGTGACATTGCTGTTCCACTAATCTCCCACCTTAACCGACGTACGGGC
TTGGTATCTCCCTAAGAGA
GCCTGAAATGTACCCTATGGCATGCTACTGTACCTGTAGTCCCGCAAGGAC
AAGTACCGCGAAATACGTA
CCACACCCCAAATGCGTCTCGCGTTCGATGATGGCACTCTCCTGCTCGAAG
ACGCGCCCAATTCAGTTCC
CTACGCGGACTGGGATGACCGCGTCGAC

Halobacterium salinarum strain R1 clone 10 genomic sequence

GenBank: AY640305.1

GenBank FASTA

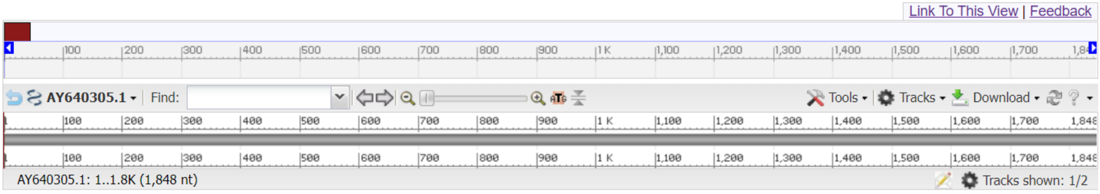

Supplement: Supplementary file 1 — Additional file 1. [file 12866_2022_2489_MOESM1_ESM.pdf]
